# Orphan drug policy analysis in China

**DOI:** 10.3389/fphar.2024.1278710

**Published:** 2024-06-13

**Authors:** Meilin Liu, Yanqin Lu, Junfeng Li, Yongtao Zhang, Shanshan Zhang, Yisheng Liu

**Affiliations:** ^1^ Key Laboratory for Biotech-Drugs of National Health Commission, Biomedical Science College and Shandong Medicinal Biotechnology Centre, Shandong First Medical University and Shandong Academy of Medical Sciences, Ji’nan, Shandong, China; ^2^ Department of Endocrinology and Metabology, The First Affiliated Hospital of Shandong First Medical University and Shandong Provincial Qianfoshan Hospital, Ji’nan, Shandong, China

**Keywords:** China, rare diseases, orphan drugs, policies, accessibility, affordability, research and development

## Abstract

Rare diseases have various types, low incidence rates, complex conditions, and are often difficult to diagnose. Due to China’s large population, there is a significant number of rare disease patients, but there is a shortage of orphan drugs. Consequently, these patients often find themselves in a situation where necessary medications are either unavailable or unaffordable. To address this urgent clinical need, China has implemented a series of orphan drug policies aimed at improving drug accessibility and affordability. In terms of drug accessibility, companies are encouraged to expedite drug development through the implementation of tax incentives, guidance for clinical research on rare diseases, and the provision of data protection periods of 6 years, along with market exclusivity periods limited to a maximum of 7 years. Moreover, exemptions for clinical trials, acceptance of overseas clinical trial data, and the creation of a list prioritizing clinically urgent new drugs from overseas have been introduced to expedite the drug registration application, review, inspection, and approval processes. In terms of drug affordability, the import value-added tax on rare disease drugs has been reduced by 3%, and various provinces and cities have established a representative rare disease protection model, which includes special funds, medical assistance programs, and serious disease insurance. The national medical insurance catalog has been adjusted to reduce the financial burden on rare disease patients, resulting in an increase in the number of orphan drugs covered by the catalog to 95 as of March 2024. By comparing orphan drug policies in the United States, the European Union, Japan, Australia, and other countries (or regions), we will provide relevant suggestions to further improve orphan drug policies in China, thus bringing more treatment options and hope to patients with rare diseases.

## 1 Introduction

Rare diseases, also known as “orphan diseases,” are characterized by their low prevalence. Although individual rare diseases affect a small number of patients, the overall impact is substantial due to the existence of numerous types of rare diseases. Currently, there are approximately 6,000 to 8,000 diseases classified as rare, with an additional 250 to 280 new diseases being identified each year (Dawkins et al., 2018). Globally, between 260 and 450 million people are affected by rare diseases, often resulting in chronic illness, disability, and premature death (Nguengang Wakap et al., 2020; Marwaha et al., 2022).

Orphan drugs, also referred to as rare disease drugs, are medications used for the prevention, treatment, and diagnosis of rare diseases or conditions (Zhang et al., 2022). However, China currently lacks a clear definition or criteria for orphan drugs. In the United States, orphan drugs are defined as drugs developed under the 1983 Orphan Drug Act, which apply to diseases affecting less than 200,000 people in the country (Sharma et al., 2010). Similarly, the European Union defines orphan drugs as those used for rare diseases with a prevalence of less than 0.5‰ and in the absence of any existing treatments (Zhang et al., 2022). Japan’s orphan drug regulations, established in 1993, categorize orphan drugs as treatments for serious diseases affecting fewer than 50,000 patients (incidence less than 0.04%). These diseases should be medically urgent and have a high likelihood of successful development. In Japan, orphan drugs are utilized when there are no alternative treatment methods available or when the safety and effectiveness of the drug surpass those of existing clinical drugs. The application for orphan drug approval in Japan requires a detailed product development plan and scientific evidence supporting the drug’s marketing (Sharma et al., 2010). Lastly, in Australia, orphan drugs are used to treat diseases or conditions that affect fewer than 2,000 people at any given time (Sharma et al., 2010).

In 2021, the United Nations adopted its first resolution on addressing the challenges faced by people living with rare diseases and their families. The resolution called on the Member States to provide safe and affordable healthcare services (The Lancet Global, 2024). Although the prevalence and incidence of rare diseases are low, China’s large population base means that there is a significant number of rare disease patients in the country (Zhang, 2024). In recent years, China has made significant efforts in this area. The State Council issued the Thirteenth Five-Year National Food Safety Plan and the Thirteenth Five-Year National Drug Safety Plan in 2017, which included policies on the prevention and treatment of rare diseases; drug research and development; drug registration, production, and distribution; and medical care for patients (The State Council, 2017b).

In order to strengthen the prevention and treatment of rare diseases, it was decided to set up an expert committee on the diagnosis, treatment, and protection of rare diseases. In 2019, the guidelines for rare disease treatment were issued, and the registration of rare disease diagnosis and treatment information was initiated by the General Office of the National Health Commission (General Office of the National Health Commission, 2019a; General Office of the National Health Commission, 2019b). Additionally, the national rare disease diagnosis and treatment collaboration network office was established in 2020 (General Office of the National Health Commission, 2020).

Currently, China lacks sufficient research and development (R&D) capabilities and the ability to imitate and produce orphan drugs. The existing drugs are expensive, making it difficult for patients with rare diseases to access them (Yang and Zhang, 2020). In contrast, out of the 160 innovative drugs approved by the US Food and Drug Administration (FDA) between 2018 and 2020, 86 were orphan drugs. However, only 27 orphan drugs were among the 153 innovative drugs approved by the National Medical Products Administration (NMPA) (Yang and Zhang, 2020; Zhang et al., 2022). To address this issue and ensure equal medical rights for patients with rare diseases, China has implemented a series of policies to promote the R&D of orphan drugs.

This paper examines China’s orphan drug policies from 2012 to the present by searching six websites, including the National Medical Products Administration, National Health Commission, National Development and Reform Commission, Ministry of Human Resources and Social Security, Ministry of Health, and Ministry of Finance. Additionally, relevant journals on orphan drug policies were searched and cited as references using literature search engines like PubMed, CNKI, and Wanfang Data Knowledge Service Platform.

## 2 Accessibility of orphan drugs

### 2.1 Policy on orphan drug R&D in China

To further enhance the reform of the pharmaceutical industry and expedite the production of drugs for patients, the National Medical Products Administration and the National Health Commission have implemented various incentive policies on orphan drugs. Some of these policies include financial subsidies for research, tax concessions, guidance on conducting clinical research related to rare diseases, a 6-year data protection period, and a market exclusivity period of no more than 7 years (National Medical Products Administration office, 2018; National Medical Products Administration, 2022). These policies aim to encourage enterprises to accelerate their efforts in drug R&D (Table 1).

**TABLE 1:** Current national policy on improving the accessibility of orphan drugs.

Issue time	Issuing agency	Policy and regulation	Content related to rare diseases
January 2009	Former China Food and Drug Administration	Notice on Printing and Distributing the Provisions on the Administration of Special Examination and Approval of New Drug Registration (Former China Food and Drug Administration, 2009)	The application for registration of new drugs for the treatment of rare diseases is subject to special examination and approval
January 2012	The State Council	Notice on Issuing the Twelfth Five-Year Plan for National Drug Safety (The State Council, 2012)	Encourage the development of orphan drugs and suitable dosage forms for children
February 2013	Former China Food and Drug Administration	Opinions on Deepening the Reform of Drug Evaluation and Approval and Further Encouraging Drug Innovation (Former China Food and Drug Administration, 2013)	Give priority to and speed up the review of orphan drugs
November 2015	Former China Food and Drug Administration	Announcement on Several Policies of Drug Registration Review and Approval (Former China Food and Drug Administration, 2015)	The application for registration of orphan drugs will be queued separately to speed up the review and approval
February 2017	General Office of the State Council	Several Opinions of the General Office of the State Council on Further Reforming and Improving Policies for Drug Production, Circulation, and Use (The General Office of the State Council, 2017a)	Conduct classification review and approval for medications related to rare diseases, children, elderly individuals, emergency use, and traditional Chinese medicine
March 2018	General Office of the State Council	Opinions on Reforming and Perfecting the Supply Guarantee and Use Policy of Generic Drugs (The General Office of the State Council, 2018a)	Encourage imitation of orphan drugs
April 2018	National Medical Products Administration office	Publicly Solicit Opinions on the Implementation Measures for the Protection of Drug Test Data (National Medical Products Administration office, 2018)	Give orphan drugs a 6-year data protection period
May 2018	National Medical Products Administration, National Health Commission	Announcement on Optimizing the Examination and Approval of Drug Registration (National Medical Products Administration and National Health Commission, 2018)	For orphan drugs that have been listed overseas, if it is considered that there is no racial difference through research, clinical trial data obtained overseas may be submitted.
June 2018	National Health Commission, etc.	Notice on Publishing the First Batch of Rare Diseases Catalog (National Health Commission et al., 2018)	The first batch of rare disease catalogs contained 121 rare diseases.
July 2018	National Medical Products Administration	Notice on Issuing the Technical Guiding Principles for Accepting Overseas Clinical Trial Data of Drugs (National Medical Products Administration, 2018)	If the application for registration of orphan drugs is partially accepted after evaluation, the data can be collected for evaluation after the drug is listed
November 2018	Center For Drug Evaluation, NMPA	Notice on Publishing the First Batch of Overseas New Drugs Urgently Needed in Clinic (Center For Drug Evaluation, 2018)	Forty kinds of overseas drugs urgently needed in clinic
May 2019	Center For Drug Evaluation, NMPA	Notice on Publishing the Second Batch of Overseas New Drugs Urgently Needed in Clinic (Center For Drug Evaluation, 2019)	Twenty-six kinds of overseas new drugs urgently needed in clinic
August 2019	National Medical Products Administration	Drug Administration Law of the People’s Republic of China (National Medical Products Administration, 2019)	Encourage the development of new drugs for rare diseases and give priority to the review and approval of orphan drugs.
September 2019	General Office of National Health and Wellness Commission, etc.	Notice on printing and distributing the first batch of encouraged generic drugs (General Office of National Health and Wellness Commission et al., 2019)	There are seven orphan drugs among 33 generic drugs
January 2020	National Medical Products Administration	Notice on the Guiding Principles for Publishing Real World Evidence to Support Drug R&D and Evaluation (Trial) (National Medical Products Administration, 2020a)	RWS and RWE are used to support the expansion of indications for orphan drugs that have been commercialized
January 2020	State Administration for Market Regulation	Management Method of Drug Note Book (State Administration for Market Regulation, 2020)	The time limit for the review of orphan drugs that are urgently needed in clinic that have been listed overseas but not listed in China is 70 days
November 2020	Center For Drug Evaluation, NMPA	Notice on Issuing the List of the Third Batch of Overseas New Drugs in Urgent Clinical Need (Center For Drug Evaluation, 2020)	Seven kinds of overseas new drugs urgently needed in clinic
December 2021	Ministry of Industry and Information Technology, etc.	Notice on Printing and Distributing the Development Plan of Pharmaceutical Industry in the Fourteenth Five-Year Plan (Ministry of Industry and Information Technology et al., 2021)	Implement support policies such as R&D expenses, additional deduction, and simple collection of value-added tax on orphan drugs
January 2022	Center For Drug Evaluation, NMPA	Notice on Issuing the Technical Guiding Principles for Clinical R&D of Drugs for Rare Diseases (Center For Drug Evaluation, 2022)	It provides suggestions and references for the R&D of orphan drugs and scientific experimental design
May 2022	National Medical Products Administration	Publicly Solicit Opinions on the Regulations for the Implementation of the Drug Administration Law of People’s Republic of China (revised draft for comments) (National Medical Products Administration, 2022)	For new drugs for rare diseases approved for marketing, the market exclusive period shall not exceed 7 years at the longest
June 2022	National Medical Products Administration, National Health Commission	Notice on Printing and Distributing the Work Plan for Temporary Import of Drugs Needed in Clinic and the Work Plan for Temporary Import of Clobazam (National Medical Products Administration and National Health Commission, 2022)	Clearly include rare disease treatment drugs in the scope of temporary import of clinically urgently needed drugs
September 2023	National Health Commission, etc.	Notice on Publishing the Second Batch of Rare Diseases Catalog (Guo Wei Yi Zheng Fa [2023] No.26) (National Health Commission et al., 2023)	The second batch of rare diseases catalog includes 86 rare diseases

In 2012, the State Council issued a notice called the “Twelfth Five-Year Plan” for national drug safety. This notice clearly stated the encouragement of R&D of orphan drugs (The State Council, 2012). Additionally, in 2018, the opinions on reforming and improving the supply guarantee and use policy of generic drugs were released. This policy also proposed the encouragement of the production of drugs for the treatment of rare diseases (The General Office of the State Council, 2018a). Furthermore, in 2019, the National Health Commission published the first batch of the “Catalog of the First Batch of Encouraged Generic Drugs,” which included 33 generic drugs. Among these drugs, seven were orphan drugs. The orphan drugs were nitisinone capsules, glatiramer acetate injection, pyridostigmine bromide tablets, treprostinil injection, bosentan tablets, icatibant acetate injection, and deferasirox dispersible tablets (General Office of National Health and Wellness Commission et al., 2019).

Moving on to 2020, the National Medical Products Administration issued the Guiding Principles for Real-world Evidence to Support Drug Development and Review (Trial) in January 2020. In addition, in August 2020, they released the Technical Guidelines for Real-world Research in Support of Pediatric Drug Development and Review (Pilot) (National Medical Products Administration, 2020a; National Medical Products Administration, 2020b). Both of these documents proposed the use of real-world study (RWS) and real-world research (RWR) to support the indication of already marketed drugs for rare diseases. Then, in 2021, the “Fourteenth Five-Year Plan” Pharmaceutical Industry Development Plan notice was released by the Ministry of Industry and Information Technology et al. This circular implemented supporting policies, including additional deduction of R&D costs and simplified collection of value-added tax on drugs for rare diseases (Ministry of Industry and Information Technology et al., 2021).

In 2022, the Statistical Guidelines for Clinical Studies of Drugs for Rare Diseases (Trial) were published. These guidelines provided guidance and recommendations on the design and statistical analysis of clinical studies for rare diseases. They also encouraged pharmaceutical companies to develop drugs for the treatment of rare diseases to improve the efficiency and quality of clinical R&D (Center For Drug Evaluation, 2022). As a result of these efforts, the number of orphan drugs under research in China has significantly increased from 2017 to 2022, with an average annual growth rate of 34% (Chen et al., 2023).

In the opinions of the Office of the National Medical Products Administration on Publicly soliciting the Implementation Measures for Drug trial Data Protection (Interim) released in 2018, it was proposed that specific drugs for rare diseases should have a data protection period of 6 years from the date when the indication was first approved in China (National Medical Products Administration office, 2018). Additionally, the implementation of Regulations of the Drug Administration Law of the People’s Republic of China, published in 2022 (Draft Amendment for comment), proposed that a new drug for rare diseases approved for marketing should be granted a maximum market monopoly period of not more than 7 years on the condition that the holder of the drug marketing authorization guarantees the drug supply. Furthermore, during this period, no other similar drugs will be approved for the market (National Medical Products Administration, 2022). All of these efforts contribute to the promotion of research and development of orphan drugs. Zhaoke Pharmaceutical Co., Ltd. is the company that develops the highest number of orphan drugs in China. Some examples of the drugs they develop include riluzole oral suspension, sodium benzoate particles, and treprostinil injection. Additionally, there are three drugs available in the domestic market for the treatment of idiopathic hypogonadotropic hypogonadism and Kallmann syndrome. These drugs are developed by Chinese companies and are gonadorelin for injection, menotropins for injection, and chorionic gonadotrophin for injection (Zhi et al., 2023).

China’s orphan drug R&D started late, and many policies are still being optimized. There are still some differences between China and the United States, the European Union, Japan, and other countries (or regions) in terms of the status of drug R&D for rare diseases. The number of approved orphan drugs in China is lower than the average level of those of the United States and the European Union. From 2018 to 2022, the National Medical Products Administration approved a total of 48 rare disease treatment drugs for the treatment of 24 rare diseases (Zhi et al., 2023). In comparison, the Food and Drug Administration (FDA) approves an average of 49 new drugs every year, of which 26 are orphan drugs, accounting for 50% of the total (Yang et al., 2023). Similarly, the European Medicines Agency (EMA) approves an average of 54 new drugs per year, with 19 being orphan drugs, accounting for about 35% (Yang et al., 2023).

### 2.2 Policy on the approval of orphan drugs in China

China has issued a series of policies to speed up the approval process for orphan drugs while still ensuring their safety and effectiveness. Exemptions from certain clinical trials and acceptance of overseas clinical trial data are available (Former China Food and Drug Administration, 2017; General Office of the State Council, 2017). Moreover, government agencies provide additional resources to expedite the registration application, evaluation, and approval for those included in the scope of priority review (National Medical Products Administration and National Health Commission, 2018). A specific channel for the review and approval of orphan drugs was established for the overseas orphan drugs with urgent clinical needs (National Medical Products Administration and National Health Commission, 2022).

In 2009, the Special Approval Management Provisions for New Drug Registration released by the former China Food and Drug Administration introduced special approval for new drug registration application for rare diseases (Former China Food and Drug Administration, 2009). In 2013, the former China and Drug Administration proposed prioritizing and expediting the review and approval of innovative drugs for the prevention and treatment of rare diseases (Former China Food and Drug Administration, 2013). Moreover, in 2015, several policies regarding drug registration review and approval were announced, introducing a separate queue for innovative drug registration applications for rare diseases and other conditions to accelerate the process (Former China Food and Drug Administration, 2015). The General Office of the State Council released opinions in 2017 that reviewed and approved the classification of rare diseases, children, the elderly, emergency drugs, and traditional Chinese medicine (General Office of the State Council, 2017a).

Since 2018, three batches of the list of clinically urgent overseas new drugs have been published, consisting of 73 drugs, with 54 of them being orphan drugs, accounting for 74% of the total (Center For Drug Evaluation, 2018; Center For Drug Evaluation, 2019; Center For Drug Evaluation, 2020). In 2020, the “Measures for the Administration of Drug Registration” clearly stated that the review time limit for clinically urgent overseas and unlisted rare disease drugs is 70 working days (State Administration for Market Regulation, 2020). Furthermore, the 2022 notice on the issuance of the Work Plan for the Temporary Import of Clinically Urgent Drugs clarified that rare disease treatment drugs are included in the scope of temporary import. If the imported drugs are used for the treatment of rare diseases, the lead import institution should be a medical institution within the national Rare Disease diagnosis and Treatment Collaboration network. The application should include a summary of nationwide drug demand, a list of medical institutions authorized to use the drug, and a letter of commitment (National Medical Products Administration and National Health Commission, 2022).

In 2018, the National Medical Products Administration and the National Health Commission issued an announcement on optimizing the review and approval of drug registration. According to this announcement, for rare disease drugs that have already been listed overseas, the applicant for import drug registration can submit the overseas clinical trial data directly for drug listing registration. This proposal was made on the condition that the applicant believes that there is no racial difference after conducting research (National Medical Products Administration and National Health Commission, 2018). In the same year, another circular was issued, which provided technical guidelines for accepting overseas clinical trial data on drugs. This circular specifically addressed rare disease drug registration applications. It stated that the overseas clinical trial data would be partially accepted, and further efficacy and safety data could be collected for evaluation after the drug is marketed (National Medical Products Administration, 2018).

In August 2019, the newly revised Drug Supervision and Administration Law of the People’s Republic of China was passed by the Standing Committee of the National People’s Congress (National Medical Products Administration, 2019). This law introduced a green channel mechanism for priority review and accelerated approval of orphan drugs based on evidence from foreign clinical trials (Zhang et al., 2019; Chen et al., 2023). Currently, some orphan drugs in China are imported. For example, Novartis Pharmaceutical has developed drugs such as fingolimod hydrochloride capsules, siponimod tablets, and ofatumumab injection, which have been listed in China in recent years for the treatment of multiple sclerosis. Bayer Pharmaceutical has also developed drugs such as riociguat tablets, iloprost, and interferon β-1b (Zhi et al., 2023).

In contrast to the mainland, Taiwan implemented the Rare Disease and Orphan Drug Act in 2000. This act is designed to enhance the availability, production, and research of products for rare diseases, thereby improving patient access to necessary medications (Sharma et al., 2010). To implement the Act, the Ministry of Health has established a Rare Disease and Orphan Drug Review Committee. In Taiwan, the “Rare Disease Drug Project Application Measures” have been issued specifically for orphan drugs. These measures simplify the registration and approval process and offer waivers for the listing application fee and registration approval fee (Department of Health, 2012).

### 2.3 Foreign incentive policy for orphan drug R&D and approval

Compared to China, some countries have specific rare disease policies aimed at R&D and marketing of drugs for rare diseases. These policies focus on improving the consistency and efficiency of the drug review process by simplifying it. In 1982, the FDA established the Orphan Drug Development Office, which is responsible for evaluating and certifying orphan drugs (Sharma et al., 2010). The Orphan Drug Act provides a range of incentive policies for the recognized orphan drugs, including a 25% tax credit for clinical research expenses, exemption from NDA/BLA application fees, access to special research funds, special approval channels, exemption from certain clinical data requirements, and 7-year market exclusivity after approval (Library of Congress, 2019; Mikami, 2019). Over time, the Orphan Drug Act has undergone five major revisions, which have added provisions for the definition of rare diseases, adjusted the period of market exclusivity for orphan drugs, increased orphan drug review fees, waived product and site fees for the prescription drugs or biological products for rare diseases, and introduced clinical trial tax credits (Library of Congress, 2007; Li et al., 2020). The act outlines a two-step process for orphan drug registration, which involves “identification” followed by marketing approval. Additionally, the FDA has established expedited review channels, shortening the average review time from 13 months to 6 months for orphan drugs with priority review status (Xin et al., 2013; Zhou et al., 2021). During the clinical trial stage, companies can request accelerated reviews for orphan drugs and can supplement clinical data after the drugs are approved for marketing. The fast track process allows clinical data for orphan drugs to be submitted on a rolling basis and facilitates communication between companies and the FDA to improve the design of more efficient clinical trials for orphan drug approval (Zhang et al., 2022).

The EU Orphan Drug Management Regulation, established in 1999, provides a clear legal framework for identifying and managing orphan drugs in the EU (Access to European Union law, 1999). It grants orphan drug status to eligible drugs and offers support for research and development, regulatory cost reduction, and a 10-year period of market exclusivity protection. In 2000, the EMA created the Committee for Orphan Medicinal Products (COMP), which is responsible for reviewing and providing opinions on orphan drugs for marketing approval. The final decision is then taken by the European Commission based on the review opinions. The EMA’s marketing review process includes both centralized and accelerated reviews. According to the Orphan Drugs Regulation, all orphan drugs in the EU must go through the centralized review procedure (He and Tian, 2014). However, innovative drugs with significant public health benefits may be submitted to the COMP for an accelerated review process, with the application being submitted at least 2–3 months prior to the marketing authorization application. If the drug qualifies for accelerated review, the review time limit is reduced from 210 to 150 days (Zhang et al., 2022).

In 1993, Japan amended the Pharmaceutical Law to address orphan drugs, including the qualification criteria, guaranteeing research and development funding for enterprises, and tax reductions (Song and Kokudo, 2013). The Orphan Drug Guidelines provide clarity on the procedures for eligibility, research recommendations, tax benefits, grants, and incentives such as a 10-year market exclusivity period (Zheng, 2023). Japan’s orphan drug registration system follows a two-step model, similar to that of the United States. The Ministry of Health, Labor, and Welfare of Japan (MHLW) is responsible for identifying orphan drugs, approving new drugs for marketing, and conducting post-marketing reviews (Ministry of Health, Labor, and Welfare of Japan, 2012). Companies first submit information to MHLW to demonstrate that the drug meets the orphan drug criteria. MHLW’s Pharmaceutical Affairs and Food Sanitation Council (PAFSC) then reviews the data and provides comments for MHLW’s consideration. MHLW makes the final decision on whether to grant orphan drug designation. The second step is market approval, where MHLW allows drugs with orphan drug status to receive priority approval and only pay reduced user fees. These fees include those for review, consultation, and approval. Data show that since the implementation of the special registration system, the approval time for orphan drug registration has been reduced by nearly 1 year compared to the registration of rare disease drugs before the policy was implemented and by nearly 2 years compared to the review of non-orphan drugs (Kiyoshi et al., 2022).

Australia has a two-step process for orphan drug registration. This process is overseen by the Therapeutic Goods Administration (TGA). First, the pharmaceutical company submits the relevant data and application for orphan drug status to the TGA. The TGA then passes this information along to the Drug Safety and Evaluation Branch (DSEB), which houses the clinical evaluation panel. It is this panel’s responsibility to review the data and determine whether the orphan drug status can be granted. Once a drug is approved, it becomes eligible for the priority approval policy during the marketing registration phase. The DSEB conducts a technical review of all relevant information and data. The TGA ensures that the work for priority approval is completed within 255 working days. In addition to this, the TGA waives all registration fees associated with the orphan drug. This includes the listing application fee, registration approval fee, and annual registration fee (Australian Government and Department of Health and Aging Therapeutic Goods Administration, 2004).

## 3 Affordability of orphan drugs

### 3.1 Pricing policy for orphan drugs

Drug prices are a matter of concern for people’s well-being and are of long-term importance to various stakeholders, including the government, patients, and enterprises. Higher drug prices can reduce the patients’ access to medications, while lower prices may discourage innovative drug research and development, ultimately affecting the access to drugs in the future (Cao, 2021). Therefore, maintaining a reasonable level of drug pricing is an important objective of drug price management. Since China implemented the Notice on the Issuance of Opinions on Promoting Drug Price Reform in 2015, orphan drugs have been exempted from government-set prices, resulting in improved drug procurement mechanisms and contributing to the control of medical insurance costs. The actual price of the drugs is primarily determined by market competition (National Development and Reform Commission et al., 2015). For drugs covered by medical insurance, such as levocarnitine oral solution, rosuvastatin calcium tablets, and bromocriptine mesilate tablets, the medical insurance department and the relevant agencies establish procedures, criteria, methods, and rules for determining the payment standards for medical insurance drugs. They also explore the development of a mechanism to guide the fair pricing of drugs. For drugs like iloprost that are not covered by medical insurance, manufacturers and distributors independently set prices based on the production costs, operational expenses, and market supply and demand.

Regarding imported drugs, in 2018, the Tariff Commission of the State Council issued a notice on the adjustment plan for the provisional import and export tariff rates in 2019. This notice includes rare disease drug raw materials such as penicillamine, pirfenidone, riluzole, and bosentan, which will have a zero tariff (The State Council, 2018b). Additionally, in 2019, 2020, and 2022, the Ministry of Finance and other departments successively promulgated the “Notice on Value-added Tax Policy for Rare Disease Drugs,” which released three batches of lists for rare disease drugs, adjusted tax policies, and imposed a reduced import value-added tax rate of 3%. They also released 21, 14, and 19 rare disease drug preparations, respectively (Ministry of Finance et al., 2019; Ministry of Finance, 2020; Ministry of Finance, 2022). In Taiwan, rare drugs are mainly imported or imitated from abroad, and they have low development costs and high success rates. The pricing of rare drugs can be moderately increased, but they are regulated by the administrative department in Taiwan (Zhong et al., 2014).

In the United States, the Orphan Drug Act grants pharmaceutical companies significant autonomy in pricing orphan drugs, allowing them to set prices based on market competition. Rare drugs enjoy a 7-year market monopoly, during which the pharmaceutical companies can set prices at their discretion. This has resulted in high prices for some rare drugs in the United States (Thamer et al., 1998). In Japan, rare drugs are considered to have limited market value, and their prices are subject to preferential pricing by the National Health Insurance. Rare drugs can also receive a 5% increase in usefulness and a 10%–20% increase in marketability (Tao et al., 2002).

Drug prices are an important factor in determining drug costs, and they are closely related to the control of drug expenses. There are significant differences in drug pricing policies among countries worldwide. In the United States, rare disease drug prices are determined solely by market competition. In China, on the other hand, there is no unified pricing management for rare disease drugs. Like other drugs, China has transitioned from government macro-control to the establishment and improvement of a market-oriented mechanism for determining drug prices. This approach helps the government understand the changing landscape of drug prices, promotes transparency in drug price information, encourages enterprises to adapt their business practices to market changes, and ultimately fosters the development of reasonable drug prices.

### 3.2 Health insurance for orphan drugs in China

China’s current medical insurance system consists of medical insurance for urban workers, basic medical insurance for urban residents, new rural cooperative medical care, and urban and rural medical assistance and serious disease pooling, which realizes the universal medical insurance (Ding, 2024). However, for the protection of drugs for rare diseases, China has not issued a national level of protection policy or system design, but it encourages all localities to explore in a pilot way (Table 2). In 2012, China’s Ministry of Health and other departments issued the notice on the 2012 New Rural Cooperative Medical Care Work, which mentioned that 12 diseases, such as chronic myelogenous leukemia and hemophilia, will be included in the pilot scope of serious illness protection (Ministry of Health et al., 2012). The Opinions on Improving the Medical Insurance and Assistance System for Major and Serious Diseases promulgated in 2021 will explore the establishment of a drug guarantee mechanism for rare diseases and integrate medical security, social assistance, charitable assistance, and other resources in accordance with the level of economic and social development and the affordability of all parties and implement comprehensive security (General Office of the State Council, 2021). As of March 2024, there are 95 orphan drugs included in the national health insurance catalog (Table 3), of which 13 are classified as class-A medical insurance (class-A medical insurance drugs are fully reimbursed) and 82 kinds are classified as class-B medical insurance (class-B medical insurance drugs are partially reimbursed) (National Medical Insurance Bureau and Ministry of Human Resources and Social Security, 2019; National Medical Insurance Bureau and Ministry of Human Resources and Social Security, 2020; National Medical Insurance Bureau and Ministry of Human Resources and Social Security, 2021; National Medical Insurance Bureau and Ministry of Human Resources and Social Security, 2023a; National Medical Insurance Bureau and Ministry of Human Resources and Social Security, 2023b). In various provinces and cities, the government actively explores the guarantee mechanism for rare disease drugs and provides protection for orphan drugs not included in the medical insurance catalog, which alleviates the burden of some rare disease patients to a certain extent.

**TABLE 2:** Current national policy on improving the affordability of orphan drugs.

Issue time	Issuing agency	Policy and Regulation	Content related to rare diseases
May, 2012	Ministry of Health, etc	Notice on doing a good Job in the new rural cooperative medical system in 2012 (Ministry of Health et al., 2012)	Twelve diseases such as hemophilia are prioritized for inclusion in the pilot scope of major disease protection
November, 2019	National Medical Insurance Bureau, Ministry of Human Resources and Social Security	Notice on Inclusion of Drugs Negotiated in 2019 in Category B of the National Drug List of Basic Medical Insurance, Work Injury Insurance and Maternity Insurance (National Medical Insurance Bureau and Ministry of Human Resources and Social Security, 2019)	In 2020, 12 orphan drugs have been added to the medical insurance catalog
December, 2019	Zhejiang Provincial Medical Insurance Bureau	Notice on the Establishment of Drug Guarantee Mechanism for Rare Diseases in Zhejiang Province (Zhejiang Provincial Medical Insurance Bureau, 2019)	The “special fund” model implements provincial-level overall planning for medical insurance for rare diseases
March, 2020	Foshan Municipal People's Government Office	Notice on Printing and Distributing the Measures for Medical Assistance in Foshan City (The People's Government of Foshan Municipality, 2020)	In the "medical assistance" mode, rare diseases are included in special assistance measures
December, 2020	National Medical Insurance Bureau, Ministry of Human Resources and Social Security	National Drug List of Basic Medical Insurance, Work Injury Insurance and Maternity Insurance (2020) (National Medical Insurance Bureau and Ministry of Human Resources and Social Security, 2020)	In 2020, seven orphan drugs were added to the medical insurance catalog
December, 2020	Shandong Province Medical Security Bureau	Notice on Further Improving the Serious Illness Insurance System in Our Province (Shandong Province Medical Security Bureau and Shandong Provincial Development and Reform Commission, 2020)	Three rare diseases, Gaucher's disease, Fabray's disease, and glycogen storage disease II, are included in the major illness insurance model
November, 2021	General Office of the State Council	Opinions on Improving the Medical Insurance and Assistance System for Serious and Serious Diseases (General Office of the State Council, 2021)	Explore the establishment of a rare disease medication guarantee mechanism; integrate resources such as medical security, social assistance, and charitable assistance; and implement comprehensive guarantees
December, 2021	National Medical Insurance Bureau, Ministry of Human Resources and Social Security	National Drug List of Basic Medical Insurance, Work Injury Insurance and Maternity Insurance (2021) (National Medical Insurance Bureau and Ministry of Human Resources and Social Security, 2021)	In 2021, eight orphan drugs were added to the medical insurance catalog
January, 2023	National Medical Insurance Bureau, Ministry of Human Resources and Social Security	National Drug List of Basic Medical Insurance, Work Injury Insurance and Maternity Insurance (2022) (National Medical Insurance Bureau and Ministry of Human Resources and Social Security, 2023b)	In 2022, seven orphan drugs were added to the medical insurance catalog
December, 2023	National Medical Insurance Bureau, Ministry of Human Resources and Social Security	National Drug List of Basic Medical Insurance, Work Injury Insurance and Maternity Insurance (2023) (National Medical Insurance Bureau and Ministry of Human Resources and Social Security, 2023a)	In 2023, 15 orphan drugs were added to the medical insurance catalog

**TABLE 3:** Orphan drugs included in the medical insurance catalog.

Number	Orphan drug	Rare disease	Medical insurance type (edition)
1	Hydrocortisone	21-Hydroxylase deficiency and congenital adrenal hypoplasia	Class-A (2004)
2	Bromocriptine mesilate tablets	Acromegaly	Class-B (2004)
3	Octreotide acetate microspheres for injection		Class-B (2017)
4	Lanreotide acetate sustained-release injection (a pre-filled syringe)		Class-B (2020)
5	Riluzole tablets	Amyotrophic lateral sclerosis	Class-B (2017)
6	Edaravone injection		Class-B (2020)
7	Riluzole oral suspension		Class-B (2022)
8	Compound vitamin B tablets	Biotinidase deficiency	Class-B (2004)
9	Levocarnitine oral solution	Carnitine deficiency and beta-ketothiolase deficiency	Class-B (2009)
10	Infliximab for injection	Crohn disease	Class-B (2017)
11	Agalsidase alfa concentrated solution for infusion	Fabry disease	Class-B (2021)
12	Avapritinib	Gastrointestinal stromal tumor	Class-B (2023)
13	Eliglustat tartrate capsules	Gaucher’s disease	Class-B (2023)
14	Siltuximab for injection	Castleman disease	Class-B (2023)
15	Pyridostigmine bromide tablets	Generalized myasthenia gravis	Class-A (2004)
16	Efgartigimod alfa injection		Class-B (2023)
17	Spesolimab injection	Generalized pustular psoriasis	Class-B (2023)
18	Eculizumab injection	Generalized myasthenia gravis, atypical hemolytic uremic syndrome, and paroxysmal nocturnal hemoglobinuria	Class-B (2023)
19	Desmopressin acetate	Hemophilia	Class-A (2004)
20	Human prothrombin complex		Class-B (2004)
21	Recombinant human coagulation factor Ⅷ		Class-B (2009)
22	Human coagulation factor Ⅷ		Class-A (2017)
23	Recombinant human coagulation factor IX		Class-B (2017)
24	Recombinant human coagulation factor VIIa		Class-B (2017)
25	Human coagulation factor Ⅸ		Class-B (2021)
26	Penicillamine tablets	Hepatolenticular degeneration	Class-A (2004)
27	Sodium dimercaptosuccinate		Class-A (2004)
28	Dimercaptosuccinic acid		Class-A (2004)
29	Zinc sulfate		Class-B (2009)
30	Danazol capsules	Hereditary angioedema	Class-B (2004)
31	Icatibant acetate injection		Class-B (2021)
32	Lanadelumab injection		Class-B (2022)
33	Potassium aspartate and magnesium aspartate	Hereditary hypomagnesemia	Class-B (2004)
34	Rosuvastatin calcium tablets		Class-B (2009)
35	Evolocumab injection		Class-B (2021)
36	Ezetimibe tablets	Homozygous hypercholesterolemia and sitosterolemia	Class-B (2017)
37	Deutetrabenazine tablets	Huntington disease	Class-B (2020)
38	Tetrabenazine tablets	Huntington disease	Class-B (2023)
39	Tafamidis meglumine	Idiopathic cardiomyopathy	Class-B (2021)
40	Calcitriol oral solution	Hypophosphatemic rickets	Class-B (2023)
41	Gonadorelin for injection	Idiopathic hypogonadotropic hypogonadism and Kallmann syndrome	Class-B (2004)
42	Menotropins for injection		Class-B (2004)
43	Chorionic gonadotrophin for injection		Class-A (2004)
44	Bosentan tablets	Idiopathic pulmonary arterial hypertension	Class-B (2017)
45	Riociguat tablets		Class-B (2017)
46	Macitentan tablets		Class-B (2017)
47	Selexipag tablets		Class-B (2017)
48	Ambrisentan tablets		Class-B (2020)
49	Treprostinil injection		Class-B (2022)
50	Pirfenidone capsules	Idiopathic pulmonary fibrosis	Class-B (2017)
51	Nintedanib esilate soft capsules		Class-B (2020)
52	Lamotrigine tablets	Lennox–Gastaut syndrome	Class-B (2009)
53	Desferrioxamine mesylate for injection	Mediterranean anemia	Class-A (2004)
54	Deferasirox dispersible tablets		Class-B (2017)
55	Baclofen tablets	Multiple sclerosis	Class-B (2004)
56	Teriflunomide tablets		Class-B (2017)
57	Fingolimod hydrochloride capsules		Class-B (2020)
58	Siponimod tablets		Class-B (2020)
59	Fampridine		Class-B (2021)
60	Dimethyl fumarate enteric capsules		Class-B (2022)
61	Ofatumumab injection		Class-B (2022)
62	Ozanimod hydrochloride capsules		Class-B (2023)
63	Thalidomide	Multiple myeloma	Class-B (2004)
64	Bortezomib for injection		Class-B (2017)
65	Lenalidomide		Class-B (2017)
66	Ixazomib citrate capsules		Class-B (2017)
67	Decitabine for injection	Myelodysplastic syndrome	Class-B (2017)
68	Azacitidine for injection		Class-B (2017)
69	Ruxolitinib phosphate tablets	Myelofibrosis	Class-B (2017)
70	Pitolisant hydrochloride tablets	Narcolepsy	Class-B (2023)
71	Glibenclamide tablets	Neonatal diabetes mellitus	Class-A (2004)
72	Insulin detemir injection		Class-A (2009)
73	Selumetinib hydrogen sulfate capsules	Neurofibromatosis	Class-B (2023)
74	Inebilizumab injection	Neuromyelitis optica	Class-B (2022)
75	Satralizumab injection		Class-B (2023)
76	Miglustat capsules	Niemann–Pick disease	Class-B (2017)
77	Carbidopa and levodopa tablets	Parkinson disease (young-onset, early-onset)	Class-B (2004)
78	Amantadine hydrochloride tablets		Class-A (2004)
79	Selegiline hydrochloride tablets		Class-B (2004)
80	Levodopa tablets		Class-A (2004)
81	Pramipexole dihydrochloride tablets		Class-B (2009)
82	Ropinirole hydrochloride tablets		Class-B (2017)
83	Entacapone, levodopa, and carbidopa tablets		Class-B (2017)
84	Rasagiline		Class-B (2017)
85	Droxidopa capsules	Parkinson disease (young-onset, early-onset) and multiple system atrophy	Class-B (2017)
86	Poractant alfa injection	Respiratory distress syndrome	Class-B (2017)
87	Mecapegfilgrastim injection	Severe congenital neutropenia	Class-B (2017)
88	Daratumumab injection	Primary light chain amyloidosis	Class-B (2021)
89	Nusinersen sodium injection	Spinal muscular atrophy	Class-B (2021)
90	Risdiplam powder for oral solution		Class-B (2022)
91	Everolimus tablets	Tuberous sclerosis complex	Class-B (2017)
92	Rapamycin topical gel		Class-B (2023)
93	Human immunoglobulin (pH4) for intravenous injection	X-linked agammaglobulinemia and primary combined immune deficiency	Class-B (2009)
94	Vigabatrin powder for oral solution	West syndrome/infantile spasms syndrome	Class-B (2023)
95	Nitisinone capsules	Tyrosinemia	Class-B (2023)

In China, Zhejiang is a typical “special fund” model, which implements provincial overall planning for rare disease medical insurance. It unifies the scope of insurance, financing standards, treatment levels, diagnosis and treatment standards, and drug management and establishes a rare disease drug security fund according to the standard of 2 yuan per person per year, special account transfer, and independent accounting (Zhejiang Provincial Medical Insurance Bureau, 2019). In the “medical assistance” model represented by Foshan city, Foshan city has established a dual guarantee model of a medical assistance system and supplementary commercial medical insurance and put rare diseases into special relief measures (The People’s Government of Foshan Municipality, 2020). With Shandong as the representative of the “serious illness insurance” model, the “Notice on further improving the serious illness insurance system in our province” released in 2020 mentioned that the minimum payment standard for three rare disease special efficacy drugs such as those for Gaucher disease, Fabry disease, and glycogen accumulation disease type II, which are included in the payment scope, is 20,000 yuan, and the part of 20,000 yuan to 400,000 yuan is reimbursed 80%. The part above 400,000 yuan (including) will be reimbursed 85%. The maximum payment limit is 900,000 yuan (Shandong Province Medical Security Bureau and Shandong Provincial Development and Reform Commission, 2020). Qingdao, Shandong province, is at the forefront in China in the construction of a rare disease medical security system. In 2005, three rare diseases, multiple sclerosis, Behçet’s disease, and Myasthenia gravis, were included in the scope of serious diseases in the medical insurance outpatient department of employees. In 2012, the General Office of the Qingdao Municipal People’s Government issued the Opinions on Establishing a Serious Disease Medical Assistance System in Urban Areas (trial implementation), which began to implement the serious disease medical assistance system. Some rare diseases were included in the scope of medical assistance for serious diseases (General Office of Qingdao Municipal Government, 2012), the supplementary medical insurance system was implemented in 2017, and the reimbursement ratio of special drugs reached 80%. The active exploration of various provinces and cities has supplemented the insufficient protection of basic medical insurance for expensive drugs outside the rare disease list, and it also provides practical cases for formulating special protection policies for rare diseases at the national level.

Compared to mainland China, Taiwan uses two approaches to alleviate the medical burden on rare disease patients. One is to pay for the treatment costs of rare disease patients under the National Health Insurance Act, and the other is to allocate a budget through central agencies, as stipulated in Article 33 of the Rare Diseases Prevention and Treatment Act. Budgets are made through central agencies to subsidize medical costs for rare diseases not covered by the Universal Health Insurance Act (Ni, 2015).

### 3.3 Foreign health insurance for orphan drugs

In the United States, the insurance subsidies for rare diseases and rare drugs are mainly in the form of commercial insurance and are supplemented by government healthcare plans. The Orphan Drug Act clearly stipulates that the commercial medical insurance in the United States has no right to deny the insurance wishes of patients with rare diseases. In addition, the United States has a personal burden of rare drugs and a lifetime benefit limit (usually 1 million US dollars). Patients with rare diseases who qualify for income requirements but do not purchase insurance can obtain medicines through the Emergency Drug Access Program (China Medical Insurance Research Association, 2014). The EU encourages member states to improve the medical reimbursement system and medical insurance and establish a special guarantee fund for rare disease drugs. In terms of the insurance system, the policies of each member state are somewhat different. For example, Belgium has a “special solidarity fund” for the reimbursement of diseases and drugs that meet specific criteria, such as clinical necessity, lack of alternative therapy, rare specific therapy, or high cost of treatment (Zhong et al., 2014). Japan has a universal medical insurance system, which is covered by the National Health Insurance Law for all Japanese nationals and is subsidized in varying proportions by the state and local governments. Since drugs not included in the medical insurance list cannot be reimbursed, Japanese patients generally do not buy drugs outside the medical insurance list, resulting in a very small market capacity for drugs outside the medical insurance list. Therefore, in Japan, almost all drugs are included in the medical insurance catalog, and the price is set by the government (Guo et al., 2010). The Australian government established the Drug Benefit Scheme in 1948, and 80% of the drug costs are covered by the drug benefit scheme. However, some expensive and effective special drugs cannot be included in the drug benefit scheme, and such drugs are included in the special guarantee scheme of “Life-saving drug project” (Guan et al., 2015).

## 4 Conclusion and future prospects

In Europe, the United States, and other regions, a relatively comprehensive policy system for rare diseases has been established. The orphan drug registration and approval system primarily follows the mode of identification and marketing approval. Relevant policy provisions for rare diseases and orphan drugs can be found on the websites of their respective drug authorities. In contrast, the concept of orphan drugs in China is not clear. Currently, the first batch of “Rare Diseases Catalog” and the second batch of “Rare Diseases Catalog” have been jointly issued by the National Health Commission, the Ministry of Science and Technology, the Ministry of Industry and Information Technology, the State Medical Products Administration, and the State Administration of Traditional Chinese Medicine in 2018 and 2023 (National Health Commission et al., 2018; National Health Commission, 2023). The second batch of the rare disease catalog covers 83 diseases, which is a limited number, and some rare disease treatment drugs are not included in the catalog.

Furthermore, we suggest extending the data protection period for orphan drugs to 10 years. This would encourage innovative enterprises to carry out R&D in this area. Health departments and drug supervision authorities should also establish special funds to support the research and development of orphan drugs. Within the European Union, seven countries, including Germany, France, and Belgium, have established public databases on orphan drug research information (Li and Dong, 2012). EMA has collaborated with FDA and the Australian Therapeutic Products Administration to share orphan drug research information and evaluation reports, aiming to accelerate the development and marketing of orphan drugs (Yang and Zhang, 2020). China should strengthen cooperation with international orphan drug-related departments, share technical information and evaluation reports on orphan drug research and development, and address the lack of evidence in orphan drug clinical research. Additionally, China should increase public awareness of rare diseases, generate public attention, and stimulate more pharmaceutical companies to engage in orphan drug R&D.

Legislative protection is the basis of realizing the medical rights and interests of patients with rare diseases. Some countries and regions, such as the United States, the European Union, Japan, Australia, and Taiwan, China, have passed laws related to rare diseases and stipulated their own medical insurance models and implementation methods for rare diseases, while China has not yet carried out the legal identification of the definition of rare diseases. Moreover, it is essential to include rare diseases in basic medical insurance and serious illness coverage in order to alleviate the financial burden on the patients. Unfortunately, China’s basic medical insurance and serious illness pooling do not cover rare diseases. To address this issue, the United States, the European Union, Japan, and Taiwan have implemented various financing models, such as commercial insurance, social charity funding, medical assistance, and government subsidy funds. This collaborative approach helps alleviate the medical burden faced by rare disease patients. In China, the number of rare disease patients with commercial insurance is very low. The majority of patients are not enrolled in any commercial insurance plans. This is because China’s commercial medical insurance regulations allow insurance companies to independently select which diseases they cover, except for certain major diseases mandated by law (Li, 2014). Lastly, medical insurance for rare diseases involves more than just payment methods. It also depends on factors such as the standard for identifying rare diseases and the price and availability of rare drugs.

## References

[B1] Access to European Union law (1999). Regulation (EC) No 141/2000 of the European parliament and of the Council of 16 december 1999 on orphan medicinal products. Available at: https://eur-lex.europa.eu/eli/reg/2000/141/ (Accessed March 13, 2024).

[B2] Australian Government, and Department of Health and Ageing Therapeutic Goods Administration (2004). Australian regulatory guidelines for prescription medicines. Available at: https://www.tga.gov.au/sites/default/files/pm-argpm-ap12.pdf (Accessed March 14, 2024).

[B3] Cao L. D. (2021). Research on monitoring and early warning of drug price in China. Tianjin: Tianjin University School of Pharmaceutical Science and Technology.

[B4] Center For Drug Evaluation, NMPA (2018). Notice on publishing the first batch of overseas new drugs urgently needed in clinic. Available at: https://www.cde.org.cn/main/news/viewInfoCommon/21de8acd6c395746b041b2ad93eb5c43 (Accessed March 13, 2024).

[B5] Center For Drug Evaluation, NMPA (2019). Notice on publishing the second batch of overseas new drugs urgently needed in clinic. Available at: https://www.cde.org.cn/main/news/viewInfoCommon/08818b168ccc85db9a42a0f6623b5688 (Accessed March, 2024).

[B6] Center For Drug Evaluation, NMPA (2020). Notice on issuing the list of the third batch of overseas new drugs in urgent clinical need. Available at: https://www.cde.org.cn/main/news/viewInfoCommon/08818b168ccc85db9a42a0f6623b5688 (Accessed March 13, 2024).

[B7] Center For Drug Evaluation, NMPA (2022). Notice of drug evaluation center of state Food and drug administration on issuing statistical guiding Principles for clinical research of drugs for rare diseases (trial). Available at: https://www.cde.org.cn/main/news/viewInfoCommon/058e0d665b785e79b7f4f24dc1dc970c (Accessed May 24, 2023).

[B8] Chen R. Liu S. Han J. Zhou S. Liu Y. Chen X. (2023). Trends in rare disease drug development. Nat. Rev. Drug Discov. 23, 168–169. 10.1038/d41573-023-00177-8 37932437

[B9] China Medical Insurance Research Association (2014). Protection status of rare diseases in some countries and regions. Soc. Secur. China 10, 71–73.

[B10] Dawkins H. J. S. Draghia-Akli R. Lasko P. Lau L. P. L. Jonker A. H. Cutillo C. M. (2018). Progress in rare diseases research 2010-2016: an IRDiRC perspective. Clin. Transl. Sci. 11 (1), 11–20. 10.1111/cts.12501 28796411 PMC5759730

[B11] Department of Health, TAIWAN (2012). Introduction and current situation of TAIWAN rare diseases regulation. Available at: https://english.doh.gov.taipei/ (Accessed March 14, 2024).

[B12] Ding B. R. (2024). Challenges and innovative suggestions for the future development of social medical insurance system in China. Health Econ. Res. 41 (01), 20–22+28. 10.14055/j.cnki.33-1056/f.2024.01.006

[B13] Former China Food and Drug Administration (2009). Notice on the issuance of the special approval management regulations for new drug registration. Available at: http://www.gov.cn/govweb/gzdt/2009-01/09/content_1200852.htm (Accessed May 23, 2023).

[B14] Former China Food and Drug Administration (2013). Opinions of the state Food and drug administration on further encouraging drug innovation in deepening the reform of drug evaluation and approval. Available at: https://www.nmpa.gov.cn/xxgk/fgwj/gzwj/gzwjyp/20130222120001551.html (Accessed May 24, 2023).

[B15] Former China Food and Drug Administration (2015). Announcement of the State Food and Drug Administration on several policies for the review and approval of drug registration. Available at: https://www.nmpa.gov.cn/xxgk/ggtg/qtggtg/20151111120001229.html (Accessed May 24, 2023).

[B16] Former China Food and Drug Administration (2017). Announcement of the national medical products administration on soliciting opinions on the "related policies on encouraging innovation of drugs and medical devices to accelerate drug and medical device market approval procedures. Available at: https://www.nmpa.gov.cn/xxgk/ggtg/qtggtg/20170511213901140.html (Accessed May 24, 2023).

[B17] General Office of National Health and Wellness Commission, General office of Ministry of Science and Technology, General Office of Ministry of Industry and Information Technology, National Medical Products Administration Comprehensive Department, and China National Intellectual Property Administration office (2019). Notice on printing and distributing the first batch of catalogues encouraging imitation drugs. Available at: https://www.gov.cn/zhengce/zhengceku/2019-11/13/content_5451674.htm (Accessed March 17, 2024).

[B18] General Office of Qingdao Municipal Government (2012). Notice on establishing a medical assistance system for urban serious illnesses (trial). Available at: http://www.qingdao.gov.cn/zwgk/zdgk/fgwj/zcwj/zfgb/n2012_16/202010/t20201025_1752119.shtml (Accessed May 24, 2023).

[B19] General Office of the National Health Commission (2019a). Notice of the general office of the national health commission on carrying out the registration of diagnosis and treatment information for rare disease cases. Available at: http://www.nhc.gov.cn/yzygj/s7659/201910/be9343380e414adb8c8d641ae8967492.shtml (Accessed May 24, 2023).

[B20] General Office of the National Health Commission (2019b). Notice of the general office of the national health commission on issuing the diagnosis and treatment guidelines for rare diseases. 2019 Edition. Available at: http://www.nhc.gov.cn/yzygj/s7659/201902/61d06b4916c348e0810ce1fceb844333.shtml (Accessed May 24, 2023).

[B21] General Office of the National Health Commission (2020). Notice of the general office of the national health commission on the establishment of the office of the national rare disease diagnosis and treatment collaborative network. Available at: http://www.nhc.gov.cn/yzygj/s7659/202001/c07900d864b64c79aa3e5c2457e46f90.shtml (Accessed May 24, 2023).

[B22] General Office of the State Council (2017). Opinions of the general office of the central committee of the communist party of China and the general office of the state Council on deepening the reform of the evaluation and approval system and encouraging innovation in medicines and medical devices. Available at: http://www.gov.cn/gongbao/content/2017/content_5232362.htm (Accessed May 24, 2023).

[B23] General Office of the State Council (2017a). Several opinions of the general office of the state Council on further reforming and improving policies for drug production, circulation, and use. Available at: http://www.gov.cn/zhengce/content/2017-02/09/content_5166743.htm (Accessed May 24, 2023).

[B24] General Office of the State Council (2018a). Opinions of the general office of the state Council on reforming and improving the supply guarantee and use policies of generic drugs. Available at: http://www.gov.cn/gongbao/content/2018/content_5283184.htm (Accessed May 24, 2023).

[B25] General Office of the State Council (2021). Opinions of the general office of the state Council on improving the medical insurance and assistance system for serious and serious diseases. Available at: http://www.gov.cn/zhengce/content/2021-11/19/content_5651446.htm (Accessed May 24, 2023).

[B26] Guan Y. R. Xiang W. Zhang F. (2015). Australian life-saving drug plan and its enlightenment to China's medical assistance system for rare diseases. Study Health Policy China 8 (08), 51–55. 10.3969/j.issn.1674-2982.2015.08.010

[B27] Guo Y. Zhang H. L. Chen J. Yuan H. M. (2010). Research on Japanese drug price policy and its enlightenment to China. China J. Pharm. Econ. (04), 63–67.

[B28] He Y. L. Tian T. (2014). On the reference significance of European and American experience to the research and development of orphan drugs in China. China Pharm. 23 (24), 6–8.

[B29] Kiyoshi S. Hiroshi S. Seiji A. Chikako S. (2022). Lifecycle management of orphan drugs approved in Japan. Orphanet J. Rare Dis. 17 (1), 299. 10.1186/S13023-022-02456-W 35906686 PMC9336109

[B30] Li Y. (2014). Discussion on the formulation of policies related to rare diseases in China — based on the investigation and analysis of the living conditions of rare disease groups. China Soft Sci. (02), 77–89.

[B31] Li Y. C. Dong J. P. (2012). Study on the current situation of rare diseases and orphan drugs management in EU. Chin. Pharm. J. 47 (05), 395–398.

[B32] Li Z. Q. Xu W. X. Yang D. Z. Wei F. Q. Yang Y. (2020). Research on incentive system design and incentive mechanism of orphan drug R&D. Chin. Pharm. Aff. 34 (12), 1422–1430. 10.16153/j.1002-7777.2020.12.011

[B33] Library of Congress (2007). H.R.3580 - Food and drug administration amendments act of 2007. Available at: https://www.congress.gov/search?q=%7B%22source%22%3A%22legislation%22%2C%22congress%22%3A%22110%22%7D (Accessed March 13, 2024).

[B34] Library of Congress (2019). H.R.1 - an Act to provide for reconciliation pursuant to titles II and V of the concurrent resolution on the budget for fiscal year 2018. Available at: https://www.congress.gov/bill/115th-congress/house-bill/1?s=7&r=2 (Accessed March 13, 2024).

[B35] Marwaha S. Knowles J. W. Ashley E. A. (2022). A guide for the diagnosis of rare and undiagnosed disease: beyond the exome. Genome Med. 14 (1), 23. 10.1186/s13073-022-01026-w 35220969 PMC8883622

[B36] Mikami K. (2019). Orphans in the market: the history of orphan drug policy. Soc. Hist. Med. 32 (3), 609–630. 10.1093/shm/hkx098 31384102 PMC6664588

[B37] Ministry of Health, Labour and Welfare of Japan, MHLW (2012). Overview of orphan drug/medical device designation system. Available at: https://www.mhlw.go.jp/english/policy/health-medical/pharmaceuticals/orphan_drug.html (Accessed March 14, 2024).

[B38] Ministry of Finance, General Administration of Customs, State Taxation Administration, and National Medical Products Administration (2019). Notice on the value added tax policy for rare disease drugs. Available at: http://www.gov.cn/zhengce/zhengceku/2019-10/15/content_5439874.htm (Accessed May 24, 2023).

[B39] Ministry of Finance, General Administration of Customs, State Taxation Administration, and National Medical Products Administration (2020). Announcement on the issuance of the second batch of list of anti cancer drugs and rare disease drugs applicable to value added tax policy. Available at: http://www.gov.cn/zhengce/zhengceku/2020-10/09/content_5549951.htm (Accessed May 24, 2023).

[B40] Ministry of Finance, General Administration of Customs, State Taxation Administration, and National Medical Products Administration (2022). Announcement on the issuance of the third batch of anti cancer drugs and rare disease drugs subject to value added tax policy. Available at: http://www.gov.cn/zhengce/zhengceku/2022-11/22/content_5728197.htm (Accessed May 24, 2023).

[B41] Ministry of Health, Ministry of Finance, Ministry of Civil and Affairs (2012). Notice of the Ministry of health and three other departments on doing a good job in the new rural cooperative medical system in 2012. Available at: http://www.gov.cn/zwgk/2012-05/25/content_2145389.htm (Accessed May 24, 2023).

[B42] Ministry of Industry and Information Technology, National Development and Reform Commission, Ministry of Science and Technology, Ministry of Commerce, National Health Commission, Emergency Management Department (2021). Notice on printing and issuing the "fourteenth five-year plan" for the development of the pharmaceutical industry. Available at: http://www.gov.cn/zhengce/zhengceku/2022-01/31/content_5671480.htm (Accessed May 24, 2023).

[B43] National Health Commission, Ministry of Science and Technology, Ministry of Industry and Information Technology, State Drug Administration, and National Administration of Traditional Chinese Medicine (2023). Notice on publishing the second batch of rare diseases catalogue. Available at: https://www.gov.cn/zhengce/zhengceku/202309/content_6905273.htm (Accessed May 13, 2024).

[B44] National Development and Reform Commission, National Health and Family Planning Commission, Ministry of Human Resources and Social Security, Ministry of Industry and Information Technology, Ministry of Finance, Ministry of Commerce (2015). Notice on issuing opinions on promoting drug price reform. Available at: http://www.gov.cn/gongbao/content/2015/content_2901386.htm (Accessed May 24, 2023).

[B45] National Health Commission, Ministry of Science and Technology, Ministry of Industry and Information Technology, State Drug Administration, National Administration of Traditional Chinese Medicine (2018). Notice on the Publication of the First Batch of Rare Disease Catalogues. Available at: http://www.nhc.gov.cn/yzygj/s7659/201806/393a9a37f39c4b458d6e830f40a4bb99.shtml (Accessed May 24, 2023).

[B46] National Medical Insurance Bureau, and Ministry of Human Resources and Social Security Bureau (2019). Notice of the Ministry of human resources and social security of the national medical insurance Bureau on including the 2019 negotiated drugs into the Class B scope of the national basic medical insurance, work injury insurance, and maternity insurance drug catalogue. Available at: http://www.gov.cn/xinwen/2019-11/28/content_5456662.htm (Accessed May 24, 2023).

[B47] National Medical Insurance Bureau, and Ministry of Human Resources and Social Security Bureau (2020). Notice of the national medical insurance administration and the Ministry of human resources and social security on issuing the catalogue of national basic medical insurance, work injury insurance, and maternity insurance drugs. Available at: http://www.gov.cn/zhengce/zhengceku/2020-12/28/content_5574062.htm (Accessed May 24, 2023).

[B48] National Medical Insurance Bureau, and Ministry of Human Resources and Social Security Bureau (2021). Notice of the national medical insurance administration and the Ministry of human resources and social security on issuing the catalogue of national basic medical insurance, work injury insurance, and maternity insurance drugs. Available at: http://www.gov.cn/zhengce/zhengceku/2021-12/03/content_5655651.htm (Accessed May 24, 2023).

[B49] National Medical Insurance Bureau, and Ministry of Human Resources and Social Security Bureau (2023a). Notice of the Ministry of human resources and social security of the national medical insurance Bureau on printing and distributing the national drug list of basic medical insurance, work injury insurance and maternity insurance. Available at: http://www.nhsa.gov.cn/art/2023/12/13/art_53_11674.html (Accessed March 17, 2024).

[B50] National Medical Insurance Bureau, and Ministry of Human Resources and Social Security Bureau (2023b). Notice of the national medical insurance administration and the Ministry of human resources and social security on issuing the catalogue of national basic medical insurance, work injury insurance, and maternity insurance drugs. Available at: http://www.gov.cn/zhengce/zhengceku/2023-01/18/content_5737840.htm (Accessed May 24, 2023).

[B51] National Medical Products Administration (2018). Notice of national medical products administration on issuing the technical guiding Principles for accepting clinical trial data of drugs abroad. Available at: https://www.nmpa.gov.cn/yaopin/ypggtg/ypqtgg/20180710151401465.html (Accessed May 24, 2023).

[B52] National Medical Products Administration (2019). Drug administration Law of the people's Republic of China. Available at: https://www.nmpa.gov.cn/xxgk/fgwj/flxzhfg/20190827083801685.html (Accessed May 24, 2023).

[B53] National Medical Products Administration (2020a). Notice of national medical products administration drug testing center on issuing the technical guiding Principles of real-world research supporting children’s drug R&D and evaluation (Trial). Available at: https://www.nmpa.gov.cn/xxgk/ggtg/ypggtg/ypqtggtg/20200901104448101.html (Accessed March 14, 2024).

[B54] National Medical Products Administration (2020b). Notice of national medical products administration on the guiding Principles for publishing real-world evidence to support drug R&D and evaluation (trial). Available at: https://www.nmpa.gov.cn/directory/web/nmpa/xxgk/ggtg/ypggtg/ypqtggtg/20200107151901190.html (Accessed March 14, 2024).

[B55] National Medical Products Administration (2022). The comprehensive department of the state Food and drug administration publicly solicits opinions on the implementation regulations of the drug administration law of the people's Republic of China (revised draft for soliciting opinions). Available at: https://www.nmpa.gov.cn/xxgk/zhqyj/zhqyjyp/20220509222233134.html (Accessed May 24, 2023).

[B56] National Medical Products Administration, and National Health Commission (2018). Announcement of the national drug administration and the national health commission on optimizing the review and approval of drug registration. Available at: https://www.nmpa.gov.cn/xxgk/ggtg/qtggtg/20180523110601517.html (Accessed May 24, 2023).

[B57] National Medical Products Administration, and National Health Commission (2022). Notice on printing and issuing the "temporary import plan for clinically urgently needed drugs" and the "temporary import plan for clobazam. Available at: http://www.gov.cn/zhengce/zhengceku/2022-06/30/content_5698580.htm (Accessed May 24, 2023).

[B58] National Medical Products Administration office (2018). National medical products administration office has publicly solicited opinions on the implementation measures for the protection of drug test data (provisional). Available at: https://www.nmpa.gov.cn/xxgk/zhqyj/zhqyjyp/20180426171801468.html (Accessed May 24, 2023).

[B59] Nguengang Wakap S. Lambert D. M. Olry A. Rodwell C. Gueydan C. Lanneau V. (2020). Estimating cumulative point prevalence of rare diseases: analysis of the Orphanet database. Eur. J. Hum. Genet. 28 (2), 165–173. 10.1038/s41431-019-0508-0 31527858 PMC6974615

[B60] Ni H. P. (2015). Study on medical security of rare diseases. China Health Insur. (05), 18–20. 10.369/j.issn.1674-3830.2015.5.5

[B61] Shandong Province Medical Security Bureau, and Shandong Provincial Development and Reform Commission (2020). Notice on further improving the serious illness insurance system in our province. Available at: http://ybj.shandong.gov.cn/art/2020/12/14/art_113604_10138646.html (Accessed March 17, 2024).

[B62] Sharma A. Jacob A. Tandon M. Kumar D. (2010). Orphan drug: development trends and strategies. J. Pharm. Bioallied Sci. 2 (4), 290–299. 10.4103/0975-7406.72128 21180460 PMC2996062

[B63] Song P. Kokudo N. (2013). Revision of measures to combat intractable diseases in Japan: three pillars will play an even greater role in the future. Intractable Rare Dis. Res. 2 (1), 33–34. 10.5582/irdr.2013.v2.1.33 25343099 PMC4204572

[B64] State Administration for Market Regulation (2020). Measures for the administration of drug registration. Available at: http://www.gov.cn/gongbao/content/2020/content_5512563.htm (Accessed May 24, 2023).

[B65] Tao Y. Shao Y. F. Zhang C. Guo C. Lei F. (2002). Management system of rare drugs in Japan. Chin. Pharm. J. (06), 69–71.

[B66] Thamer M. Brennan N. Semansky R. (1998). A cross-national comparison of orphan drug policies: implications for the U.S. Orphan Drug Act. J. Health Polit. Policy Law 23 (2), 265–290. 10.1215/03616878-23-2-265 9565894

[B67] The Lancet Global, Health (2024). The landscape for rare diseases in 2024. Lancet Glob. Health 12 (3), e341. 10.1016/s2214-109x(24)00056-1 38365397

[B68] The People's Government of Foshan Municipality (2020). Notice of the office of foshan municipal people's government on printing and distributing the medical assistance measures of foshan city. Available at: http://www.foshan.gov.cn/zwgk/rdzt/lwlb/zcjd/content/post_4516293.html (Accessed March 14, 2023).

[B69] The State Council (2012). Notice of the state Council on issuing the national drug safety "12th five-year plan. Available at: http://www.gov.cn/zwgk/2012-02/13/content_2065197.htm (Accessed May 23, 2023).

[B70] The State Council (2017b). Notice of the state Council on printing and distributing the “13th Five-Year” National Food Safety Plan and “13th Five-Year” National drug safety plan. Available at: http://www.gov.cn/zhengce/content/2017-02/21/content_5169755.htm (Accessed May 24, 2023).

[B71] The State Council (2018b). Notice of the state Council tariff commission on the adjustment plan for provisional import and export tax rates in 2019. Available at: http://www.gov.cn/xinwen/2018-12/24/content_5351532.htm (Accessed May 24, 2023).

[B72] Xin X. X. Guan X. D. Cheng J. Shi L. W. (2013). Comparative study on registration system of orphan drugs at home and abroad. Chin. Pharm. J. 48 (15), 1323–1328. 10.11669/cpj.2013.15.023

[B73] Yang T. Zhang H. (2020). Incentive system for research and development of rare diseases drugs abroad and its enlightenment. China Pharm. 23 (08), 1612–1614+1647.

[B74] Yang Z. Y. Li Y. Ding W. X. (2023). Analysis on the R&D policy and status of rare diseases drugs in China. Drug Eval. Res. 46 (10), 2076–2082. 10.7501/j.issn.1674-6376.2023.10.003

[B75] Zhang J. Z. Song X. L. Wang Z. Z. Wang P. Yi M. (2022). Ethical enlightenment of incentive mechanism for orphan drug research and development abroad to China. Chin. Med. Ethics 35 (09), 971–977. 10.12026/j.issn.1001-8565.2022.09.08

[B76] Zhang S. Chen L. Zhang Z. Zhao Y. (2019). Orphan drug development in China: progress and challenges. Lancet 394 (10204), 1127–1128. 10.1016/s0140-6736(19)32179-8 31571591

[B77] Zhang X. Wu Q. Lan L. Peng D. Guan H. Luo K. (2024). Haplotype-resolved genome assembly of the diploid Rosa chinensis provides insight into the mechanisms underlying key ornamental traits. Health China 4 (03), 14. 10.1186/s43897-024-00088-1 PMC11020927 38622744

[B78] Zhejiang Provincial Medical Insurance Bureau (2019). Notice on establishing a drug guarantee mechanism for rare diseases in Zhejiang province. Available at: http://ybj.zj.gov.cn/art/2019/12/31/art_1229113757_601196.html (Accessed March 14, 2023).

[B79] Zheng H. Q. (2023). Study on the legal guarantee of drug accessibility for orphans with rare diseases in China. Guangzhou: Guangzhou University.

[B80] Zhi W. Liu M. L. Yang D. Zhang S. S. Lu Y. Y. Han J. X. (2023). Analysis of marketed orphan drugs in China. Intractable Rare Dis. Res. 12 (3), 132–140. 10.5582/irdr.2023.01030 37662620 PMC10468412

[B81] Zhong J. Hao W. Huo J. P. Zhao Z. G. (2014). International comparative study on medical security for rare diseases. Drug Eval. 11 (06), 8–11.

[B82] Zhou W. J. Gao Y. Wang M. Zhang X. C. (2021). A brief analysis of the research and development of orphan drugs abroad from 2010 to 2019. Proceeding Clin. Med. 30 (10), 770–773. 10.16047/j.cnki.cn14-1300/r.2021.10.014

